# Biobased Eutectic Solvents: A Natural Catch 22

**DOI:** 10.1021/acssuschemeng.5c02302

**Published:** 2025-04-08

**Authors:** Nicolas Schaeffer, João A. P. Coutinho

**Affiliations:** † CICECO - Aveiro Institute of Materials, Department of Chemistry, 56062 University of Aveiro, 3810-193 Aveiro, Portugal

**Keywords:** green solvent, sustainability, life cycle assessment, environmental impact, NADES

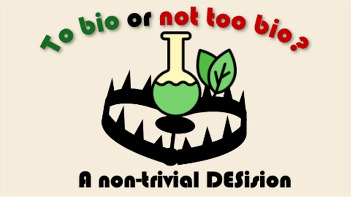

As a consequence of mitigation measures to address fossil resource depletion and global warming impacts, the chemistry sector has witnessed a diversification of solvents driven by the shift from fossil-based to biobased feedstocks.^1^ Due to the possibility of obtaining chemical precursors from renewable sources, biobased solvents are often labeled as green solvents while potentially neglecting the various complementary factors that are required to grant this designation.^2^ Chief among these is the rapidly growing field of neoteric solvents, particularly (deep) eutectic solvents (DES) and its subsets of biobased natural deep eutectic solvents (NADES). DES are defined as “eutectic mixtures of Lewis or Brønsted acids and bases” and generated significant interest at the academic level in a wide range of areas from solvent extraction to catalysis.^3^ Overlooking the issue of metastability and esterfication of certain NADES mixtures, it is now well documented through various life cycle assessment (LCA) studies that the environmental sustainability of biochemicals is dependent on the biofeedstock production and its land usage.^4–7^ While reductions in global warming impacts and fossil fuel dependence are possible particularly for biochemicals coming from biomass residues, managed forests, or fermentation, studies indicate this can comes at the expense of increased eutrophication and greater water scarcity. Such environmental trade-offs must not be overlooked when discussing sustainability, particularly given that the promotion of biomass for industry if enacted globally may result in a substantial increase in biomass demand.^8^ Such is the catch 22 pitfall that NADES and other biobased solvents must avoid, whereby a natural solvent is naturally detrimental when all factors are considered.

The ACS Green Chemistry Institute defines the ideal solvent in terms of “greeness” and one that must also meet the criteria of “scalability” and “wide utility” (https://reagents.acsgcipr.org/interpret-venn-diagrams/). These are two daunting criteria when considering that the global market for solvents is projected to reach 37.4 million metric t by 2030, with alcohols accounting for 13.5 million metric t.^9^ To place these numbers in perspective, a simple calculation is shown in Figure 1. It estimates the land area required to substitute global solvent demand by two distinct hydrophobic NADES composed of thymol+menthol (orange) or palmitic acid+menthol (blue), assuming these components are exclusively obtained from thyme, mint, and palm oils. Although Figure 1 clearly represents an argument by *reductio ad absurdum* and should not be taken at face value, it serves to illustrate the issues facing NADES in scaling to industrially relevant volumes. Furthermore, scalability implies economy of scale, except for some small organic acids, alcohols, and sugars;^7^ currently, any large scale production of chemicals is based on fossil fuels and the Haber–Bosch process (for nitrogen-bearing compounds). To avoid being trapped in niche applications due to expensive manufacturing, DES research must be willing to accept the synthetic origin of solvent precursors.

**Figure 1:**
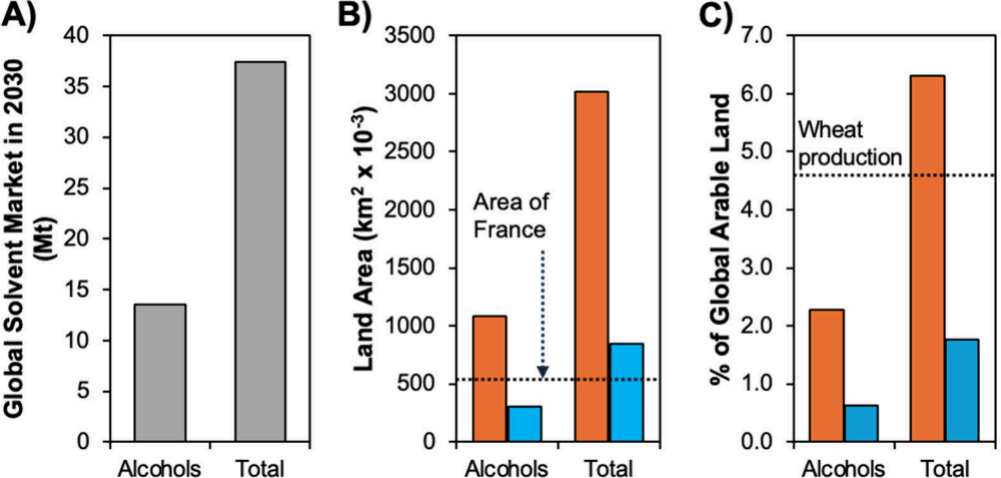
(A) Projected alcohol and total global solvent market in 2030 in million metric tons (Mt).^9^ (B) Land area required and (C) its relation to the global arable land (47.81 × 10^6^ km^2^ in 2022) to substitute the alcohol or total solvent demand in panel A by fully biosourced thymol+menthol (orange) or palmitic acid+menthol (blue) eutectic solvents for *x*_menthol_ = 0.5. The land area was estimated based on the production of 250, 150, and 3500 kg of oil per hectare per annum for mint, thyme, and palm oil, respectively, along with menthol, thymol, and palmitic acid contents of 80%, 60%, and 44%, respectively.^10–12^

What does this mean for the future of green solvents and DES? The expressed opinion does not mean to suggest that these should be abandoned; all solutions are required to address climatic challenges ahead. Biorefineries represent an integral part of the solution considering that chemical manufacturing is currently the third largest industrial subsector in terms of direct CO_2_ emissions and the first in energy consumption.^13^ Rather, this assessment serves to reinforce that natural does not automatically equate sustainable. The greenest solvent scenario is the absence of solvent. As this constitutes an impossibility for numerous applications, a solvent’s greeness is therefore intrinsically linked to its application and the existing alternatives. LCA and toxicity studies are prerogatives for sustainability, as they often provide a more nuanced description given that net zero impact chemicals across all categories are unrealistic. This fallacy is perfectly exemplified in a recent work by Bhattacharyya et al., showing that a 2 order of magnitude reduction in the carbon footprint and total variable costs was possible when substituting two “green” NADES with “dirty” H_2_SO_4_ with H_2_O_2_ for the leaching of lithium-ion battery black mass.^14^

Rather than justifying the use of DES due to their perceived, yet generally unjustified, green, nontoxic, and biodegradable nature, it is important to understand what differentiates DES from common solvents to maximize their performance and minimize their impact. Most relevant of all is the capacity of DES to overcome solubility issues of target compounds through liquefaction, be it a metal extractant, catalyst, or pharmaceutical active ingredient, extending their liquid state applicability at a desired temperature. Only through recognizing the advantages and limitations of DES can their usage be truly sustainable.
